# Expression of Prostatic Acid Phosphatase in Rat Circumvallate Papillae

**DOI:** 10.1371/journal.pone.0158401

**Published:** 2016-06-27

**Authors:** Kentaro Nishida, Teruyo Kubota, Saki Matsumoto, Junki Kato, Yu Watanabe, Atsuko Yamamoto, Mari Furui, Akihiro Ohishi, Kazuki Nagasawa

**Affiliations:** Department of Environmental Biochemistry, Kyoto Pharmaceutical University, 5 Nakauchi-cho, Misasagi, Yamashina-ku, Kyoto, Japan; German Institute of Human Nutrition Potsdam-Rehbruecke, GERMANY

## Abstract

ATP and its metabolites are important for taste signaling in taste buds, and thus a clearance system for them would play critical roles in maintenance of gustatory function. A previous report revealed that mRNAs for ecto-5′-nucleotidase (NT5E) and prostatic acid phosphatase (PAP) were expressed by taste cells of taste buds, and NT5E-immunoreactivity was detected in taste cells. However, there was no information on PAP-immunoreactivity in taste buds. In this study, we examined the expression profile of PAP in rat taste buds. In the isolated rat taste buds, we detected expression of mRNA for PAP, but NT5E was not detected differing from the case of mouse ones (Dando et al., 2012, J Neuroscience). On immunohistochemical analysis, PAP-immunoreactivity was found predominantly in NTPDase2-positive type I and SNAP25-positive type III taste cells, while there were no apparent signals of it in PLC-β2-positive type II, α-gustducin-positive type II, AADC-positive type III and 5HT-positive type III ones. As for NT5E, we could not detect its immunoreactivity in rat taste buds, and co-localization of it with any taste cell markers, although mouse taste buds expressed NT5E as reported previously. These findings suggest that PAP expressed by type I and one of type III taste cells of rats may contribute to metabolic regulation of the extracellular levels of adenine nucleotides in the taste buds of circumvallate papillae, and the regulating mechanisms for adenine nucleotides in taste buds might be different between rats and mice.

## Introduction

Taste cells receive tastant stimuli, and transmit taste information to the brain via the chorda tympani and glossopharyngeal nerves. Finger et al. (2005) revealed that ATP was an important transmitter in taste signaling in taste buds. It has been reported that sweet, bitter and umami taste stimuli cause the release of ATP into the intercellular space from type II taste cells via a hemichannel, pannexin 1, and an ion channel, calcium homeostasis modulator 1 [1, 2], and ATP transmits signals to type III taste cells and/or sensory nerve terminals via P2X2/3 receptors [3, 4]. Moreover, an ADP receptor, P2Y1, and adenosine A2b receptor are reported to be expressed in type II taste cells, suggesting that not only ATP but also its metabolites, e.g., ADP and adenosine, play important roles in gustatory signaling [5–8].

A clearance system for ATP and its metabolites is important to maintain gustatory function. In our limited knowledge, there is no report on transport system for adenine nucleotides from extracellular to intracellular space. In contrast, we reported the expression of equilibrative nucleoside transporter 1, as a molecule responsible for clearance of extracellular adenosine, in type II and III taste cells [9]. These findings clearly point out the critical roles of metabolic enzymes for adenine nucleotides as their clearance system. Among ectoenzymes, nucleoside triphosphate diphosphohydrolase 2 (NTPDase2), which metabolizes ATP into ADP [10–12], was reported to be expressed in type I taste cells [13]. Regarding adenosine-generating enzymes, Dando et al. found the expression of ecto-5′-nucleotidase (NT5E, also known as CD73) in the regions surrounding taste buds and glutamic acid decarboxylase (GAD) 1-positive type III taste cells of mice on immunohistochemistry. In addition, the expression of mRNA for prostatic acid phosphatase (PAP) was detected in *Ntpdase2*-positive type I and *Snap25*-positive type III taste cells of mice on single cell RT-PCR [7]. Although AMP can be hydrolyzed by not only NT5E but also PAP [14], expression of PAP in taste cells at protein levels and comparison of its expression profile with that of NT5E have not been unveiled yet. Therefore, in this study, we examined the localization of PAP in rat circumvallate papillae (CP) in detail.

## Materials and Methods

### Animals

Male Sprague–Dawley (SD) rats (200–300 g; Japan SLC, Hamamatsu, Japan) and male C57BL/6 mice (8-weeks old; Japan Charles River, Kanagawa, Japan) were housed with food and water available ad libitum in a controlled environment with a 12/12-h light/dark cycle. All experiments were approved by the Experimental Animal Research Committee of Kyoto Pharmaceutical University and performed according to the Guidelines for Animal Experimentation of Kyoto Pharmaceutical University.

### Exfoliation of epithelial tissues including rat CP

Rats were perfused transcardially with saline under deep anesthesia (pentobarbital sodium, 50 mg/kg, intraperitoneal (i.p.)). As reported previously, rat epithelial tissues containing CP were exfoliated from the tongue by injection of an enzyme cocktail comprising 2.5 mg/mL Dispase II (#4942078; Roche, Tokyo, Japan), 1.0 mg/mL collagenase D (#1088858; Roche), and 1.0 mg/mL trypsin inhibitor (#T9128; Sigma-Aldrich, St. Louis, MO) for 30 min at room temperature, and then the epithelial tissues were treated with RNAlater^Ⓡ^ solution (Sigma-Aldrich) at −20°C until use [15].

### Reverse transcription (RT)-polymerase chain reaction (PCR) analysis

**Fig 1:**
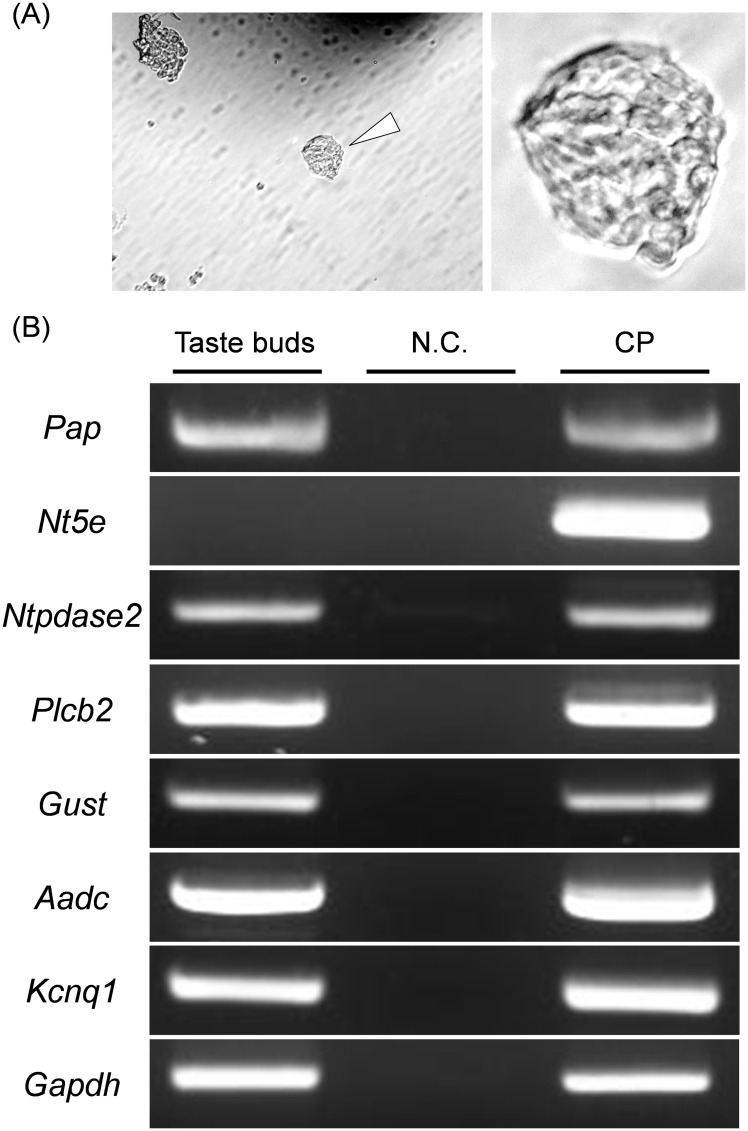
mRNA expression of PAP and NT5E in rat CP. Taste buds were isolated from digested epithelial tissues of rat CP by means of a patch pipette (A). Expression of *Pap* and *Nt5e* was examined by nested PCR (B). *Ntpdase2*, *Plcb2*, *Gust*, *Aadc and Kcnq1* served as positive controls for taste buds including taste cells. *Taste buds*, *CP* and *N*.*C*. denotes taste buds that were isolated with a patch pipette (arrowhead, A), taste bud-containing epithelium in rat CP and negative control (H_2_O), respectively.

As shown in Fig 1A, isolated taste buds were collected from the digested epithelial tissues of rat CP by means of a patch pipette using a micromanipulator (NARISHIGE, Tokyo, Japan), and directly reverse-transcribed with a CellAmp whole transcriptome amplification kit (Takara, Shiga, Japan) and Ex Taq Hot start version (Takara) according to the manufacturers’ instruction manuals, and then the cDNA-amplified products were used as templates for isolated taste buds. Total RNA in taste bud-containing epithelium of rat CP was extracted and reverse-transcribed with a NucleoSpin RNA^Ⓡ^ XS kit (Macherey-Nagel, Düren, Germany), and a PrimeScript^™^ RT reagent kit with gDNA Eraser (Takara) according to the manufacturers’ instruction manuals. Nested PCR was performed using rTaq DNA polymerase (Takara), and the primer sets, annealing temperatures, and cycle numbers were shown in Table 1. All reactions for PCR were performed with the following parameters: 94°C for 5 min; the designated cycles of 94°C for 30 s, the designated annealing temperature for 30 s, and 72°C for 30 s; and a final step at 72°C for 10 min. A negative control involving the template (H_2_O control) was set in all PCR reactions. The PCR products were confirmed by sequence analysis (data not shown).

**Table 1:** Primers for RT-PCR.

Gene (Accession No.)	PCR	Primer Sequences	Annealing temperature (°C)	Cycle numbers (cycles)	Product size (bp)
*Pap* (NM_001134901)	1^st^	F: 5’-TCTGAGGATCGGTTGCTATACCTG-3’	46	40	697
		R: 5’-GCACTTGGTGGTTGCTTGTG-3’			
	2^nd^	F: 5’-GACTGTCCTCGCTTTCAAGAACTC-3’	46	40	642
		R: 5’-CATACACTCTGTGGCCCAGT-3’			
*Nt5e* (NM_021576)	1^st^	F: 5’-CACACCAACACCTTTCTCTACACAG -3’	50	40	606
		R: 5’-CTGGCCATATCGATGCACAC-3’			
	2^nd^	F: 5’-ACCCATTCATAGTCACCTCTGACG-3’	50	40	511
		R: 5’-TTCTTCAGGGTGGACCCTTTC-3’			
*Ntpdase2* (NM_172030)	1^st^	F:5’-AGAGACAGACATGCTAGCACACCAC-3’	55	15	634
		R: 5’-TGTTGAAGAGCCGAGAGACGAG-3’			
	2^nd^	F: 5’-TTGAGGCAGTGACACAGACG-3’	55	38	506
		R: 5’-GGTAGCATTGCTGGTTCCTGAC-3’			
*Plcb2* (NM_053478)	1^st^	F: 5’-AAGGCATATCTGAGCCAAGG-3’	52	25	535
		R: 5’-TTGCAAGGTGACAGGCACTG-3’			
	2^nd^	F: 5’-GGACGATGAAACCTCAATAGCC-3’	52	40	475
		R: 5’-GGCAGCTTCTACACGTTTGC-3’			
*Gust* (NM_173139)	1^st^	F: 5’-TAGTTCAGAGAGCAAGGAGTCAGC-3’	55	15	520
		R: 5’-CCGGGAATGTAGAACGTCTTG-3’			
	2^nd^	F: 5’-AAGAACTGGAGAAGAAGCTTCAGG-3’	55	38	366
		R: 5’-AGGCTTGAATTCCTGGATCG-3’			
*Aadc* (NM_012545)	1^st^	F: 5’-CACATCTGATCAGGCACATTCCTC-3’	52	40	683
		R: 5’-TTAGCCGGAAGCAGACCAAC-3’			
	2^nd^	F: 5’-AGCAATTCCTTCAGATGGCAAC-3’	52	40	537
		R: 5’-TGAGACAGCTTCACGTGCTTTC-3’			
*Kcnq1* (NM_032073)	1^st^	F: 5’-CAGCCTCACTCATCCAGACTGC-3’	52	25	511
		R: 5’-ACATCTCGCACATCGTAGGG-3’			
	2^nd^	F: 5’-CTATGCTGCGGAGAATCCTGAC-3’	52	40	456
		R: 5’-CGTGCTTGCTGGAATTTCTTC-3’			
*Gapdh* (NM_017008.4)	1^st^	F: 5’-TCATTGACCTGAACTACATGGTC-3’	55	40	567
		R: 5’-CGTTCAGCTCTGGGATGAC-3’			

### Immunohistochemical analysis

The localization of antigens was examined by free-floating immunohistochemistry [16, 17]. SD rats and C57BL/6 mice were perfused transcardially with 4% paraformaldehyde in 0.1 M Sorensen’s phosphate buffer (pH 7.4) containing 0.2% picric acid under deep anesthesia (pentobarbital sodium, 50 mg/kg, i.p.), and then their tongues were removed. The tongues were sectioned at 40 μm thickness using a freezing microtome, and free-floating sections were immunoreacted with the primary antibodies (Table 2) in phosphate-buffered saline containing 1% normal donkey or goat serum, 0.3% Triton X-100, 0.3% bovine serum albumin (Sigma-Aldrich), and 0.05% sodium azide for 3 days at 4°C, followed by incubation with the secondary antibodies (Table 2) for 1 day at 4°C in the same buffer as that used for the primary antibodies. For all immunostaining, a negative control was prepared by omitting the primary antibodies, and the reproducibility of immunostaining was confirmed by examining sections from 3 or 4 rats per immunostaining. The sections were mounted on glass slides and then enclosed using a Prolong^®^ antifade kit (Life Technologies). Photomicrographs were obtained under a confocal laser microscope (LSM510META; Carl Zeiss, Germany). The antigen specificity of antibodies for PAP and NT5E was evaluated by means of an antibody adsorption test (S1 Fig). Single staining using the primary antibodies was performed to confirm the conjugation of Alexa Fluor^®^ to the antibodies (S2 Fig). Furthermore, for quantitative analysis of PAP expression levels, PAP-immunopositive areas merged with taste cell marker-immunoreactivity were analyzed using the Image J software (ver. 1.48v, National Institutes of Health, Bethesda, MD), and percentage of the merged area to total taste cell marker one per a taste bud was calculated.

**Table 2:** Antibodies used for immunohistochemistry.

Antigen	1 st Ab	2 nd Ab
PAP	Chicken anti-PAP Ab	Goat anti-chicken IgY conjugated with FITC
	(1:100; #PAP, Aves Labs)	(1:1000; #F-1005, Aves Labs)
NT5E(CD73)	Sheep anti-NT5E Ab	Donkey anti-sheep IgG conjugated with Alexa Fluor 594
	(1:100; #AF4488, R&D Systems)	(1:1000; #A11016, Life Technologies^™^)
NTPDase2	Sheep anti-NTPDase2 Ab	Donkey anti-sheep IgG conjugated with Alexa Fluor 594
	(1:200; #AF5797, R&D Systems)	(1:1000; #A11016, Life Technologies^™^)
PLC-β2	Rabbit anti-PLC-β2 Ab	Goat anti-rabbit IgG conjugated with Alexa Fluor 546
	(1:1000; #sc-206, Santa Cruz Biotechnology)	(1:1000; #A11010, Life Technologies^™^)
α-Gustducin	Rabbit anti-α-gustducin Ab	Goat anti-rabbit IgG conjugated with Alexa Fluor 546
	(1:200; #sc-395, Santa Cruz Biotechnology)	(1:1000; #A11010, Life Technologies^™^)
SNAP-25	Mouse anti-SNAP-25 Ab	Donkey anti-mouse IgG conjugated with Alexa Fluor 594
	(1:500; #610366, BD Biosciences)	(1:1000; #A21203, Life Technologies^™^)
		Goat anti-mouse IgG conjugated with Alexa Fluor 546
		(1:1000; #A11003, Life Technologies^™^)
AADC	Rabbit anti-AADC Ab	Goat anti-rabbit IgG conjugated with Alexa Fluor 546
	(1:50; #BML-AZ1030-0050, Enzo)	(1:1000; #A11010, Life Technologies^™^)
5HT	Rabbit anti-5HT Ab	Goat anti-rabbit IgG conjugated with Alexa Fluor 546
	(1: 500; S5545, Sigma-Aldrich)	(1:1000; #A11010, Life Technologies^™^)

## Results

### Expression levels of PAP and NT5E mRNA in isolated taste buds of rat CP

To reveal the expression of PAP and NT5E mRNA in taste buds, we carried out nested PCR. NTPDase2, PLC-β2, α-gustducin, AADC and KCNQ1 were used as positive controls for taste buds [13, 18–21]. As all of taste cell markers, PAP mRNA was found in both isolated taste buds and taste bud-containing epithelium, whereas NT5E mRNA was detected in the latter, but not the former (Fig 1B). Thus, there was a difference in expression profiles of mRNAs for PAP and NT5E.

### Localization of PAP and NT5E in rat CP

**Fig 2:**
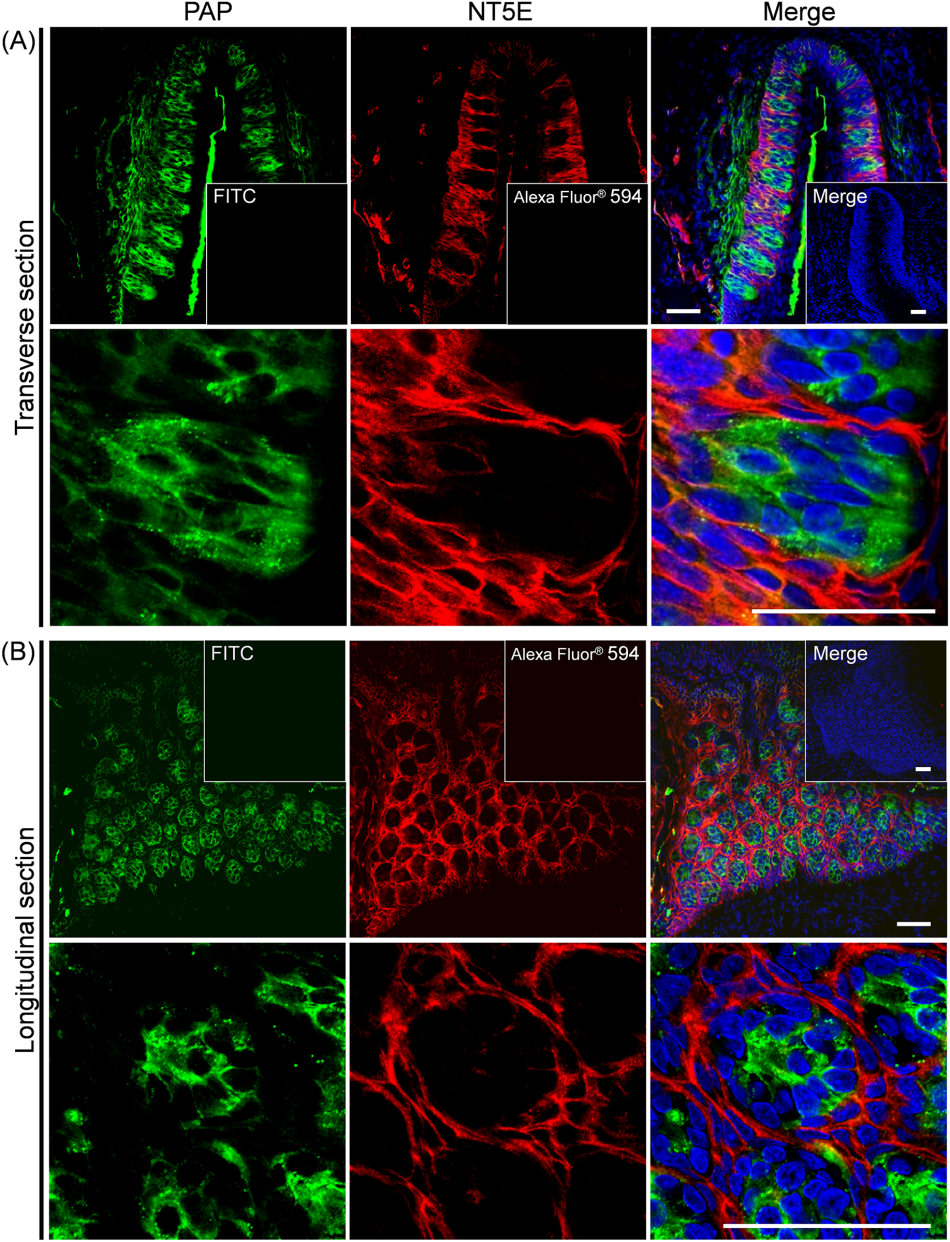
Immunohistochemical localization of PAP and NT5E in transverse and longitudinal sections of rat CP. Representative photomicrographs for double staining with PAP (green) and NT5E (red) are shown for transverse (**A**) and longitudinal (**B**) sections. The nuclei were counterstained with Hoechst 33258 (blue). In the insets, cryosections were treated with the first antibody-free solution. Immunoreactivity due to the second antibodies only was used as a negative control, as shown with Alexa Fluor^®^ 594 or fluorescein isothiocyanate (FITC). *Scale bar*, 50 μm.

**Fig 3:**
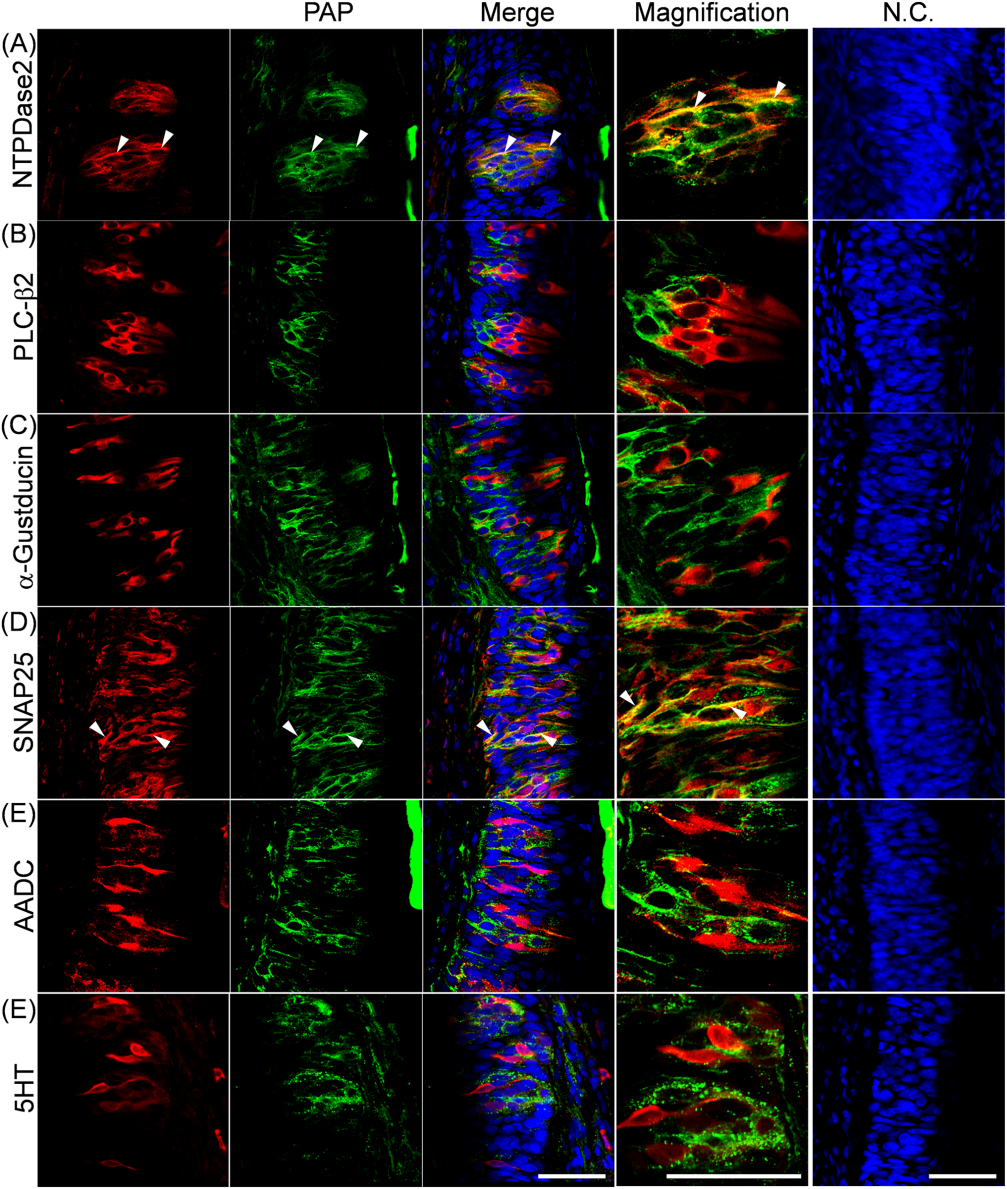
Immunohistochemical localization of PAP in transverse sections of rat CP. Representative photomicrographs for double staining with PAP (green), and the type I cell marker NTPDase2 (**A**, red), the type II cell marker PLC-β2 (**B**, red), or α-gustducin (**C**, red), or the type III cell marker SNAP25 (**D**, red), AADC (**E**, red), or 5HT (**F**, red) are shown for transverse sections. In the photomicrographs on the right, cryosections were treated with the first antibody-free solution. Immunoreactivity due to the second antibodies only was used as a negative control (N.C.). *Arrowheads* indicate the colocalization of PAP and taste cell markers. The nuclei were counterstained with Hoechst 33258 (blue). *Scale bar*, 50 μm.

**Fig 4:**
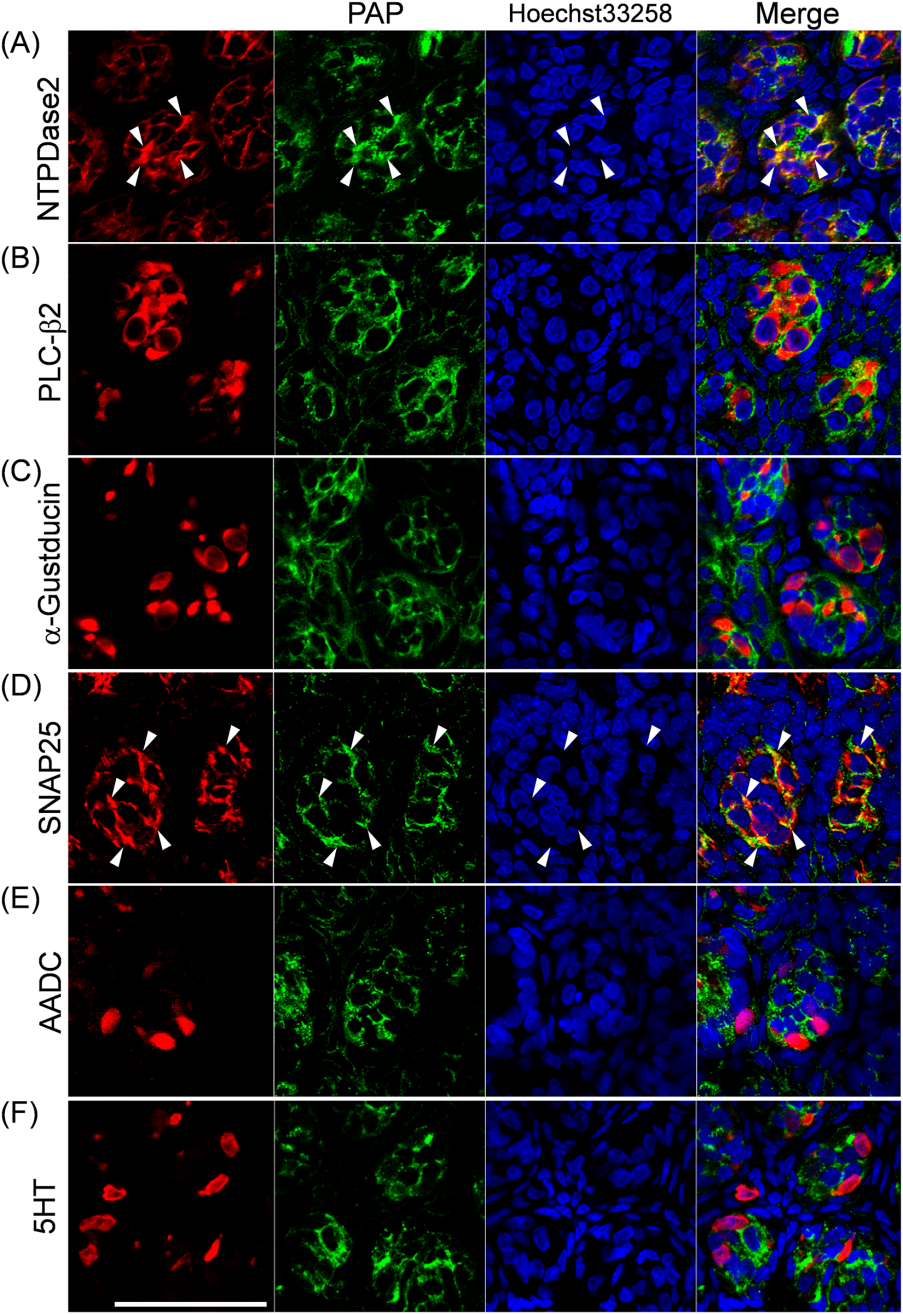
Immunohistochemical localization of PAP in longitudinal sections of rat CP. Representative photomicrographs for double staining with PAP (green), and the type I cell marker NTPDase2 (**A**, red), the type II cell marker PLC-β2 (**B**, red), or α-gustducin (**C**; red), or the type III cell marker SNAP25 (**D**, red), AADC (**E**, red), or 5HT (**F**, red) are shown for longitudinal sections. *Arrowheads* indicate the colocalization of PAP and taste cell markers. The nuclei were counterstained with Hoechst 33258 (blue). *Scale bar*, 50 μm.

As shown in Fig 2, PAP-immunoreactivity was detected exclusively in the taste buds of rat CP, while NT5E-immunoreactivity was predominantly found in the regions surrounding the taste buds of rat CP. Regarding the PAP-expressing taste cell types, its immunoreactivity was localized in NTPDase2-positive type I and SNAP25-positive type III taste cells rather than PLC-β2-positive type II, α-gustducin-positive type II, AADC-positive type III or 5HT-positive type III taste cells (Figs 3 and 4). Quantitative results on expression levels of PAP in each type of taste cells were shown in Table 3. The PAP-immunopositive area in transverse and longitudinal sections was calculated to be 21.7 ± 0.3% and 18.3 ± 7.1% of the NTPDase2-immunopositive areas, and 28.1 ± 5.4% and 33.8 ± 9.4% of the SNAP25-immunopositive areas, respectively. As for other taste cell markers, weak co-localization with PAP was found, but their levels were almost comparable with the background level, which was calculated to be 4.3 ± 5.2% as the percentage of the 5HT-immunopositive area merged with NTPDase2-immunopositive one in transverse section (Mean ± S.D., 13 taste buds evaluated) as a negative control for this quantitative analysis. Based on these findings, we judged that PAP was predominantly expressed by NTPDase2-positive type I and SNAP25-positive type III taste cells, this being consistent with expression profile of PAP mRNA as found by Dando et al [7].

**Table 3:** Quantitative analysis of co-expression of PAP and markers in taste buds of rat CP.

Marker	Percentage of indicated marker positive cells expressing PAP	
	Transverse section	Longitudinal section
NTPDase2	21.7 ± 0.3% (12)	18.3 ± 7.1% (26)
PLC-β2	6.7 ± 1.5% (17)	8.6 ± 5.7% (24)
α-Gustducin	6.4 ± 1.8% (12)	6.2 ± 5.2% (21)
SNAP25	28.1 ± 5.4% (11)	33.8 ± 9.4% (20)
AADC	6.0 ± 1.3% (12)	4.3 ± 4.4% (24)
5HT	8.1 ± 4.9% (11)	2.6 ± 3.6% (20)

Each value is based on data shown in Figs 3 and 4, and means the percentage of the PAP-immunopositive area merged with taste cell marker-immunopositive one. The numbers of evaluated taste buds from 3 or 4 rats were shown in parentheses. Mean ± S.D.

**Fig 5:**
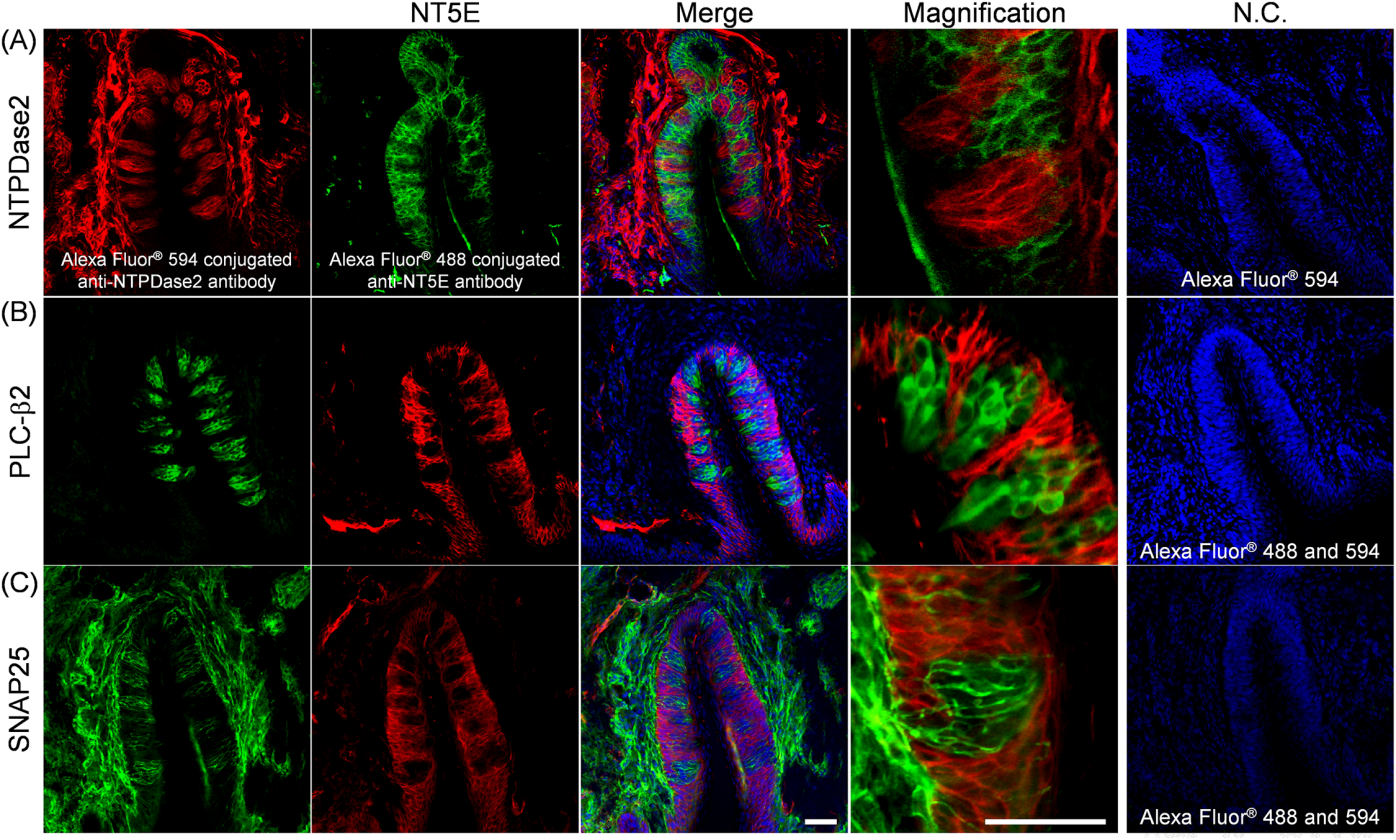
Immunohistochemical localization of NT5E in transverse sections of rat CP. Representative photomicrographs for double staining with NT5E (**A**, green; **B** and **C**, red), and the type I cell marker NTPDase2 (**A**, red), the type II cell marker PLC-β2 (**B**, green), or the type III cell marker SNAP25 (**C**, green) are shown. In panel **A**, double staining for NT5E and NTPDase2 was performed directly using Alexa Fluor^®^-conjugated primary antibodies, as described under Materials and Methods. The nuclei were counterstained with Hoechst 33258 (blue). In the photomicrographs on the right, cryosections were treated with the first antibody-free solution, and immunoreactivity due to the second antibodies only was used as a negative control (N.C.), as shown with Alexa Fluor^®^ 488 and/or 594. *Scale bar*, 50 μm.

**Fig 6:**
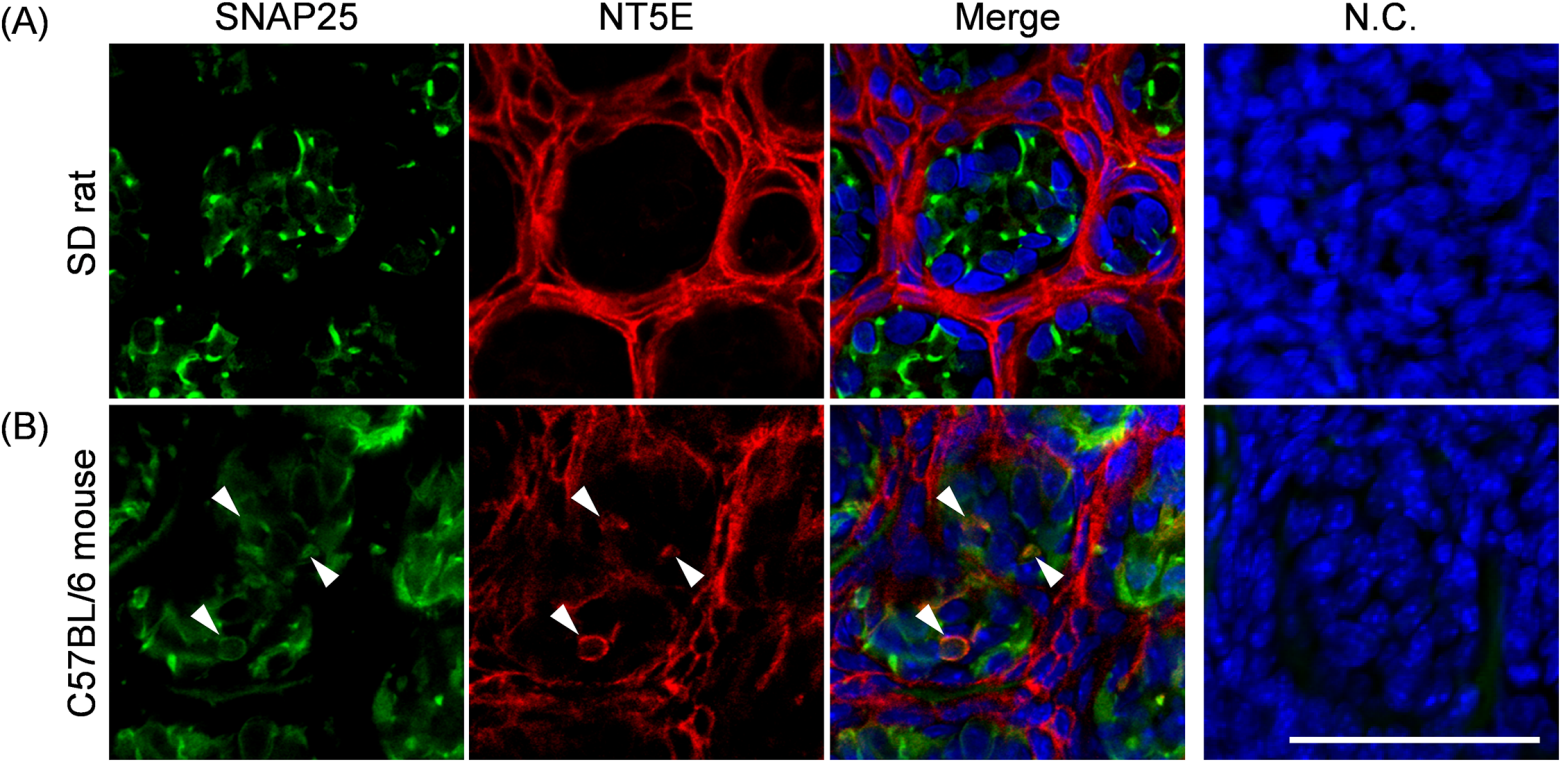
Immunohistochemical localization of NT5E in longitudinal sections of rat and mouse CP. Representative photomicrographs for double staining with NT5E (red) and type III cell marker SNAP25 (green) in longitudinal sections of SD rat (**A**) and C57BL/6 mouse (**B**) CP are shown. The nuclei were counterstained with Hoechst 33258 (blue). In the photomicrographs on the right, cryosections were treated with the first antibody-free solution. Immunoreactivity due to the second antibodies only was used as a negative control (N.C.). *Scale bar*, 50 μm.

We could not observe evident colocalization of immunoreactivity of NT5E with any taste cell markers (Fig 5), indicating that differing from the case of PAP, NT5E was not expressed by taste cells of the rat CP. Dando et al. reported that NT5E in mouse CP was expressed in not only the regions surrounding taste buds but also the GAD1-positive type III taste cells [7], there being inconsistency between rats and mice. Thus, we directly compared its expression in CP between SD rats and C57BL/6 mice. We confirmed that the antibody used for the detection of NT5E has specific immunoreactivity to both rat and mouse NT5E (S3 Fig). As shown in Fig 6, NT5E-immunoreactivity was found in both rat and mouse CP, but there was an apparent difference in the expression profiles. That is to say, in mouse CP, NT5E was expressed by SNAP25-positive type III taste cells as found by Dando et al.[7], while in rat CP it was found in the regions surrounding taste buds (Fig 6), indicating a species difference in NT5E expression between rats and mice.

## Discussion

In this study, we examined the expression profile of PAP in rat CP in detail, and obtained the following results: 1) PAP was detected in the isolated taste buds and was predominantly expressed by type I- and III-taste cells, whereas 2) NT5E was mainly found in the regions surrounding the taste buds, but not in the taste buds. These results indicated that PAP might play an important role in the metabolic regulation of extracellular levels of adenine nucleotides in the taste buds of the rat CP, and there might be a species difference in the metabolic system for adenine nucleotides between rats and mice.

Based on the literature, in taste buds under neutral pH conditions, ATP is mainly hydrolyzed into ADP by NTPDase2 [13], while there has been no report on expression of ADPase activity involved in ADP hydrolysis, although AMPase activity is exerted by NT5E [7]. In this study, we found expression of PAP in taste buds (Figs 1–4). ADP is a preferable substrate of PAP under neutral and acidic pH conditions [22], and acidification occurs in limited vicinity regions by hydrolyzation of ATP into ADP [23]. Together, it is suggested that PAP might be a responsible molecule for ADP hydrolysis in rat taste buds. Since we have no direct evidence for this at present, detail investigations are needed to demonstrate acidification in the extracellular microenvironment in the vicinity of the plasma membrane in taste buds.

Dando et al. reported that NT5E was expressed by not only type III-taste cells but also in the regions surrounding taste buds of mice, this expression profile being also confirmed in this study. However, the expression levels of NT5E was much greater the outside regions than the inside regions of mouse CP (Fig 6). Thus, we think that PAP might have greater roles than NT5E in metabolic regulation of adenine nucleotides in both mice and rats.

PAP-immunoreactivity is co-localized with NTPDase2-positive type I taste cells (Figs 3 and 4), coinciding with the findings as to expression of its mRNA [7]. On *Ntpdase2*-knockout in mice, tastant-induced taste nerve responses were suppressed compared to in the case of the wild-type mice [24], suggesting that NTPDase2-mediated ATP degradation is important for taste sensation in taste buds. Additionally, the optimum pH range for NTPDase2 is 4.5–8.5 [11] as for PAP (pH 3–8) [25]. Thus, the co-localization of PAP and NTPDase2 in type I taste cells is considered to be reasonable for an effective adenine nucleotide-metabolic cascade in the extracellular space of taste buds.

## Conclusions

We demonstrated that PAP-immunoreactivity was present in type I and one of type III taste cells of taste buds. Thus, it is suggested that PAP might be a responsible ectoenzyme for metabolism of extracellular nucleotides, being involved in the regulation of taste signaling in taste buds.

## Supporting Information

S1 Fig PAP- and NT5E-antibody adsorption test. Representative images of immunohistochemistry with PAP (**A**, green) and NT5E (**B**, red) in rat circumvallate papillae (CP) are shown (first antibody (Ab) plus second Ab). The nuclei were counterstained with Hoechst 33258 (blue). Anti-PAP and NT5E antibodies were preadsorbed with recombinant mouse PAP and NT5E, and then cryosections were treated with the preadsorbed antibodies, as shown for the first antibody adsorption. Cryosections were treated with the first antibody-free solution. Immunoreactivity due to the second antibodies only was used as a negative control (N.C.). *Scale bar*, 50 μm.
(TIF)

S2 Fig Immunohistochemical localization of NT5E in rat circumvallate papillae (CP). Double staining for NT5E (green, Alexa Fluor^®^ 488) and NTPDase2 (red, Alexa Fluor^®^ 594) was performed directly using Alexa Fluor^®^-conjugated the primary antibodies (**A**). The nuclei were counterstained with Hoechst 33258 (blue). Single staining for NT5E (**B**, green) or NTPDase2 (**C**, red) was performed to confirm conjugation of Alexa Fluor^®^ to primary antibodies. Cryosections were treated with the first antibody-free solution, and immunoreactivity due to Alexa Fluor^®^ 594-conjugated anti-sheep IgG antibodies only was used as a negative control (**D**). *Scale bar*, 50 μm.
(TIF)

S3 Fig Cross-reactivity of antibodies for NT5E. The cross-reactivity of antibodies for NT5E was examined by Western blot analysis. Rat and mouse dorsal root ganglia (DRG) were used as NT5E-expressing tissues. GAPDH was the loading control.
(TIF)

S1 Protocol (DOC)

S1 Table Antigens used for the adsorption test. (DOC)

## Abbreviations

CP: circumvallate papillae
NT5E: ecto-5′-nucleotidase
NTPDase: nucleoside triphosphate diphosphohydrolase
PAP: prostatic acid phosphatase
SNAP: synaptosomal-associated protein
5HT: serotonin
