# Supplementary figures and images for: Expression of Prostatic Acid Phosphatase in Rat Circumvallate Papillae
Source: PLoS One. 2016 Jun 27;11(6):e0158401. doi: 10.1371/journal.pone.0158401 (PMC4922667; doi:10.1371/journal.pone.0158401)

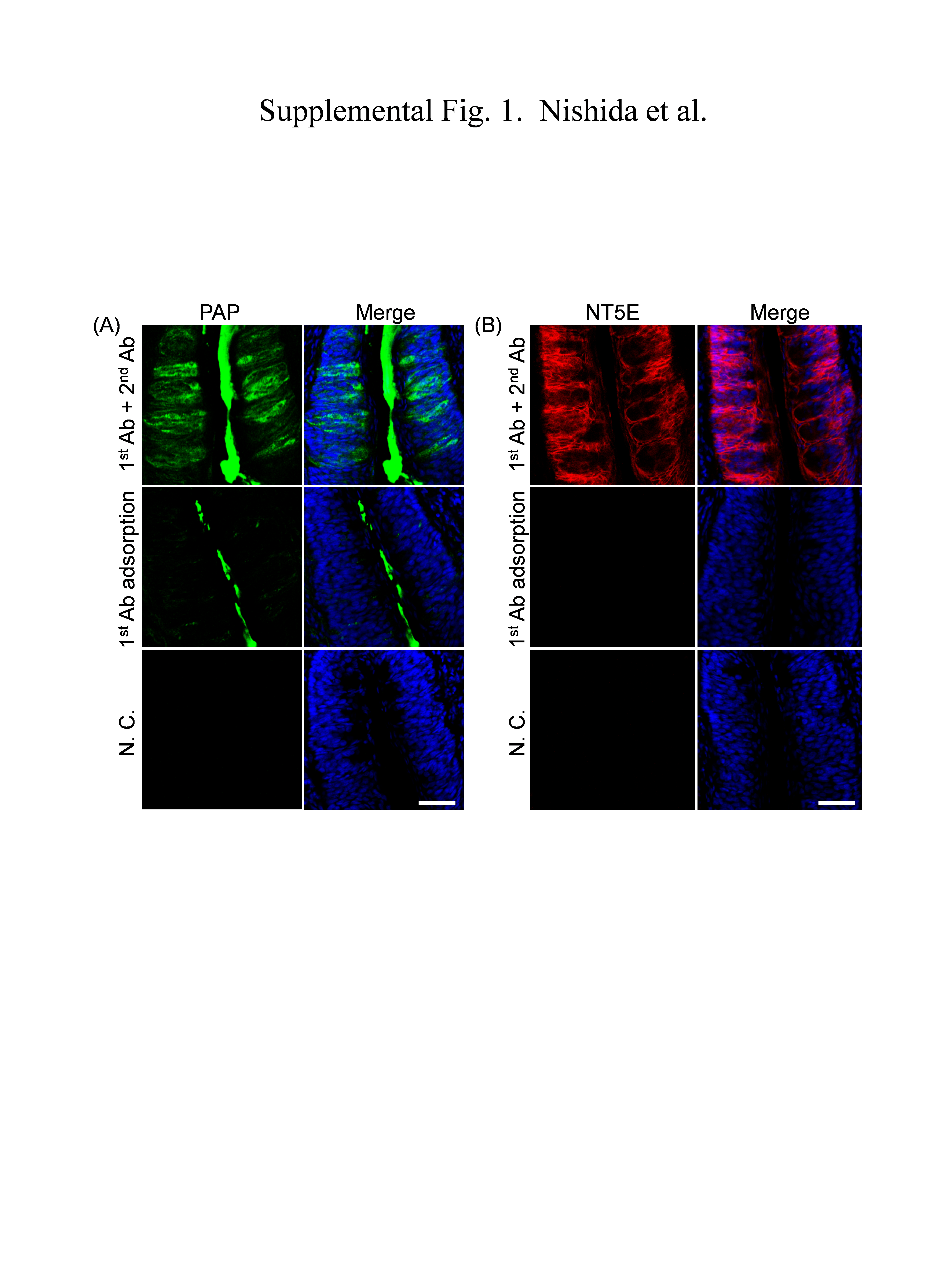

Supplement: S1 Fig — Representative images of immunohistochemistry with PAP (A, green) and NT5E (B, red) in rat circumvallate papillae (CP) are shown (first antibody (Ab) plus second Ab). The nuclei were counterstained with Hoechst 33258 (blue). Anti-PAP and NT5E antibodies were preadsorbed with recombinant mouse PAP and NT5E, and then cryosections were treated with the preadsorbed antibodies, as shown for the first antibody adsorption. Cryosections were treated with the first antibody-free solution. Immunoreactivity due to the second antibodies only was used as a negative control (N.C.). Scale bar, 50 μm. (TIF) [file pone.0158401.s001.tif]

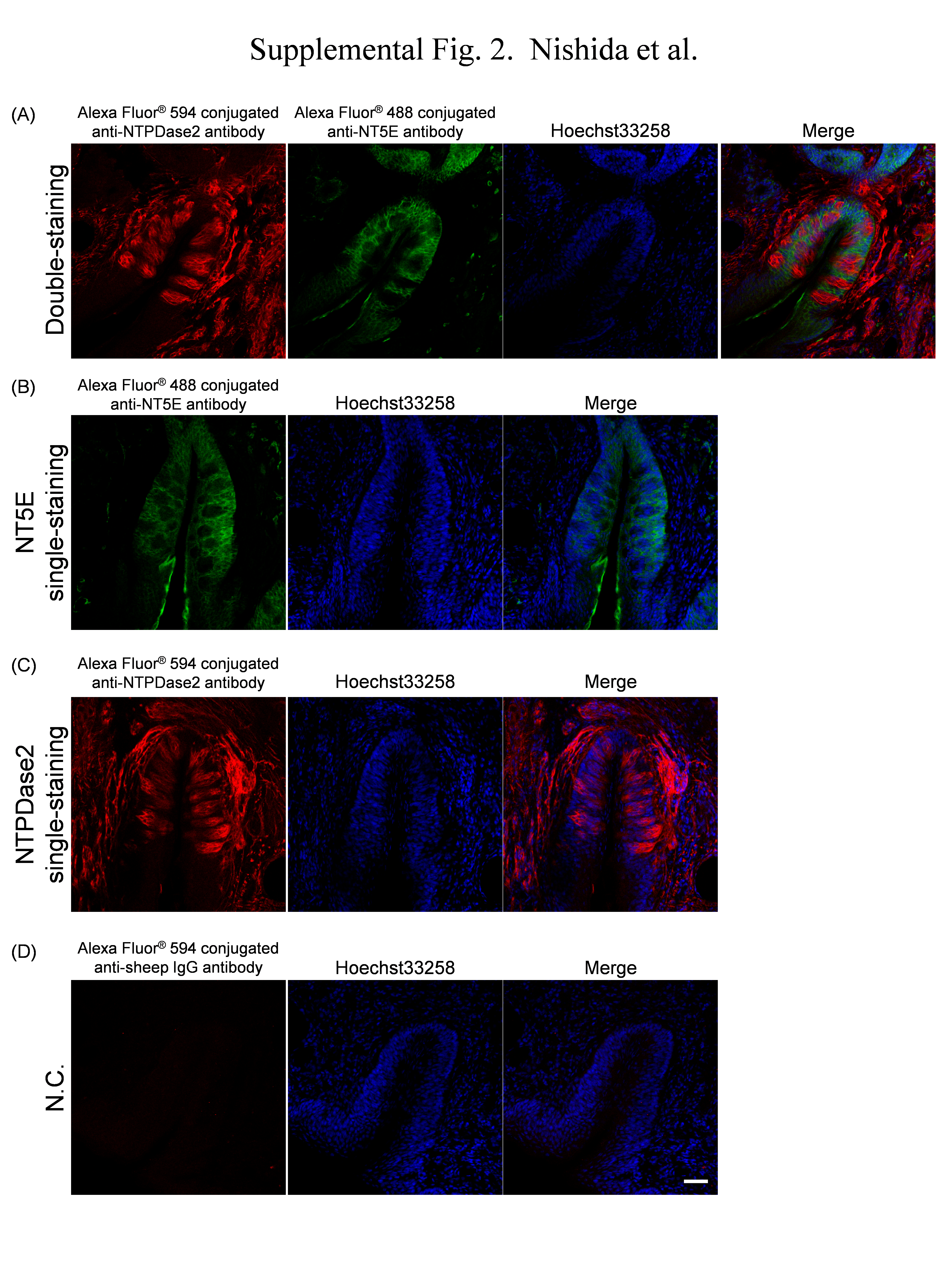

Supplement: S2 Fig — Double staining for NT5E (green, Alexa Fluor® 488) and NTPDase2 (red, Alexa Fluor® 594) was performed directly using Alexa Fluor®-conjugated the primary antibodies (A). The nuclei were counterstained with Hoechst 33258 (blue). Single staining for NT5E (B, green) or NTPDase2 (C, red) was performed to confirm conjugation of Alexa Fluor® to primary antibodies. Cryosections were treated with the first antibody-free solution, and immunoreactivity due to Alexa Fluor® 594-conjugated anti-sheep IgG antibodies only was used as a negative control (D). Scale bar, 50 μm. (TIF) [file pone.0158401.s002.tif]

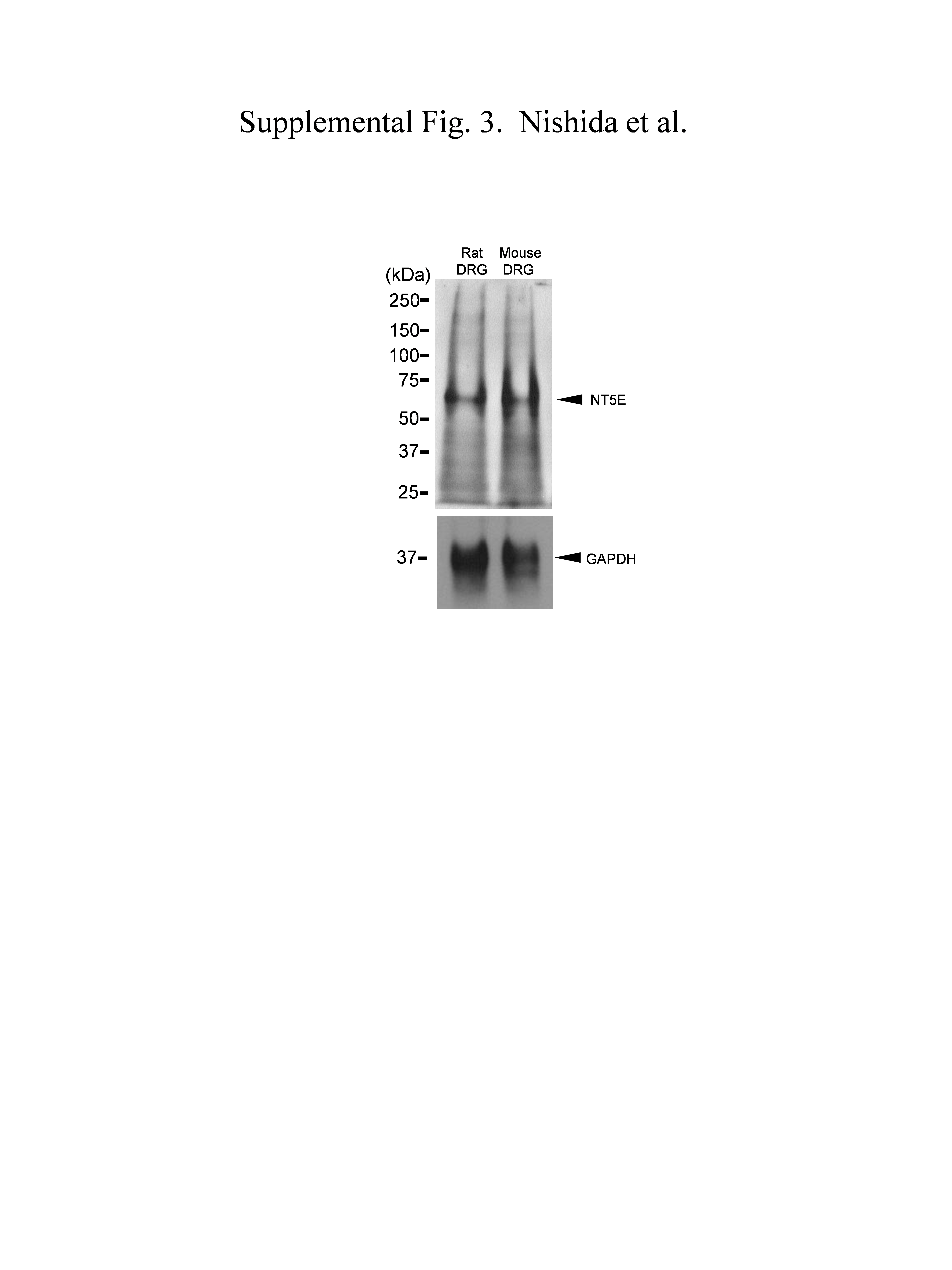

Supplement: S3 Fig — The cross-reactivity of antibodies for NT5E was examined by Western blot analysis. Rat and mouse dorsal root ganglia (DRG) were used as NT5E-expressing tissues. GAPDH was the loading control. (TIF) [file pone.0158401.s003.tif]
