# Supplementary material for: Expression of Prostatic Acid Phosphatase in Rat Circumvallate Papillae
Source: PLoS One. 2016 Jun 27;11(6):e0158401. doi: 10.1371/journal.pone.0158401 (PMC4922667; doi:10.1371/journal.pone.0158401)
Supplement: S1 Protocol — (DOC) [file pone.0158401.s004.doc]

**S1 Protocol**

**Antibody adsorption test**

The antigen specificity of antibodies was evaluated by means of an antibody adsorption test (S1 Fig). Namely, primary antibodies were adsorbed with a recombinant protein for 1 h at room temperature (S1 Table), and then cryosections were treated with the adsorbed antibodies instead of primary antibodies, followed by incubation with the secondary antibodies.

**Western blot analysis**

Samples were prepared and applied to sodium dodecyl sulfate-polyacrylamide gels at 20 μg protein/lane, and then transferred to polyvinylidene difluoride membranes (Immobilon; Merck Millipore, Billerica, MA). After blocking with Tris-buffered saline containing 0.1% Tween 20 and 5% nonfat dry milk (#9999; Cell Signaling Technology, Tokyo, Japan), the membranes were incubated overnight at 4°C with a sheep anti-NT5E antibody (1:1000, #AF4488; R&D Systems) or a mouse anti-glyceraldehyde-3-phosphate dehydrogenase (GAPDH) antibody (1:1000, #016-25523; Wako). After washing, the membranes were incubated with horseradish peroxidase-conjugated anti-sheep IgG or anti-mouse IgG antibodies (1:10,000). NT5E or GAPDH was detected using a chemiluminescent substrate (ECL Plus; GE Healthcare Japan, Tokyo, Japan). Protein concentrations were determined by the method of Bradford with bovine serum albumin as the standard.

**Conjugation of Alexa Fluor® to primary antibodies**

Double-staining for NT5E and NTPDase2 was performed by immunohistochemistry using Alexa Fluor®-conjugated primary antibodies. In brief, sheep anti-NT5E antibodies (#AF4488; R&D Systems) or sheep anti-NTPDase2 ones (#AF5797; R&D Systems) were directly conjugated with the succinimidyl ester of Alexa Fluor® 488 (#A-20000; Life Technologies) or 594 (#A-20004; Life Technologies) carboxylic acid, respectively. Single staining using the primary antibodies was performed to confirm the conjugation of Alexa Fluor® to the antibodies (S2 Fig).
