# Supplementary material for: Expression of Prostatic Acid Phosphatase in Rat Circumvallate Papillae
Source: PLoS One. 2016 Jun 27;11(6):e0158401. doi: 10.1371/journal.pone.0158401 (PMC4922667; doi:10.1371/journal.pone.0158401)
Supplement: S1 Table — (DOC) [file pone.0158401.s005.doc]

**S1 Table. Antigens used for the adsorption test.**

| Target | Antibody | Antigen for adsorption test |
| --- | --- | --- |
| NT5E | Sheep anti-CD73 Ab  (4 μg/mL; #AF4488, R&D Systems) | Recombinant mouse NT5E  (12 μg/mL; #50231-M08H, Sino Biological, Beijing, China) |
| PAP | Chicken anti-PAP Ab  (100 μg/mL; #PAP, Aves Labs) | Recombinant mouse PAP  (300 μg/mL; #51018-M08H, Sino Biological) |
